# Pyorrhoea Alveolaris

**Published:** 1916-03

**Authors:** 


**Pyorrhoea Alveolaris.** The U. S. Public Health Service reports an examination of 190 cases. 187 showed the endamoeba. 87 of these were treated with emetin but none lost it permanently. Improvement of the condition of the teeth and gums was noted in 22. 2 remained stationary and 1 became worse. While emetin is an amoebacide, it will not alone cure pyorrhoea.

